# Public health impacts of gold mining in Ghana: A scoping review of environmental exposures and intersectoral resilience strategies

**DOI:** 10.4102/jphia.v17i1.1820

**Published:** 2026-06-25

**Authors:** Perfect Edinam Asamoah, Abigail Osei Owusu

**Affiliations:** 1 School of Medicine, Unit of Lifespan and Population Health, University of Nottingham, Nottingham, United Kingdom; 2 Department of Social Sciences, Dodowa Health Research Centre, Dodowa, Ghana

**Keywords:** gold mining, artisanal and small-scale mining, environmental health exposures, public health impacts, coping and adaptation strategies, institutional responses, Ghana

## Abstract

**Background:**

Ghana’s rapid expansion of large-scale and artisanal gold mining has intensified environmental degradation and public health risks in affected communities. Although prior research has largely focused on contamination and ecosystem impacts, less is known about how these hazards are experienced in everyday life.

**Aim:**

This scoping review therefore aimed to map and synthesise evidence on the health and well-being impacts of gold mining on communities in Ghana, including community adaptation and coping responses and the actions of institutions and other stakeholders to address these impacts.

**Setting:**

The review examines evidence from communities located in gold-mining regions across Ghana.

**Methods:**

Guided by Arksey and O’Malley’s framework and the Joanna Briggs Institute (JBI) Manual, we searched PubMed, MEDLINE, and Scopus, alongside grey literature and university repositories. Screening and data charting were conducted in Microsoft Excel, and reporting followed PRISMA-ScR guidelines. Nineteen empirical studies published between 2012 and 2025 were included.

**Results:**

Reported outcomes included respiratory illness, dermatological conditions, waterborne infections, malaria, psychosocial distress, and limited evidence on reproductive health effects, linked to exposure to polluted air, contaminated water, and ecological disruption. Mining also undermined livelihoods reduced agricultural productivity, and increased food insecurity. Communities responded through livelihood diversification, informal support networks, alternative water use, and migration, although some strategies introduced new risks. Institutional responses were fragmented, small-scale, and weakly enforced.

**Conclusion:**

Gold mining generates multilayered risks affecting community health and well-being.

**Contribution:**

This study highlights the need for community based participatory research, integrated environmental health surveillance and stronger regulatory enforcement to mitigate mining-related public health harms.

## Introduction

Gold mining is a major driver of Ghana’s economy, contributing substantially to employment, rural livelihoods and national revenue.^1^ In 2024 alone, gold exports were valued at $11.6 billion, representing approximately 57% of the country’s total export earnings.^2^ Ghana now ranks among the world’s leading gold producers, with rapid growth in both large-scale industrial mining and artisanal and small-scale mining (ASM), including informal and illegal operations commonly referred to as *Galamsey*.^3^ The rapid expansion of gold mining has led to widespread ecological degradation with direct implications for public health.^4^ Soils and waterways are contaminated with mercury, arsenic, cadmium and lead, while rivers suffer from sedimentation and turbidity. Extensive deforestation, land degradation and airborne particulate matter from blasting, ore processing and untarred roads further exacerbate environmental stress.^5^ These environmental changes create multiple pathways of human exposure: communities rely on contaminated water sources, inhale toxic dust and face increased vector-borne disease risks in abandoned pits, all compounded by the loss of ecosystem services that support health and nutrition.^6,7^

Beyond these physical hazards, the growing affluence of both foreign and local miners has been associated with heightened engagement in unsafe sexual practices, contributing to elevated rates of sexually transmitted infections and unplanned pregnancies.^8^ Competition over land and water, coupled with low compensation and unresolved settlement disputes between mining companies and local communities, generates long-term social tensions and conflict.^9,10^ Where reclamation of any of these lands occurs, they are often infertile as a result of mining activities, undermining agricultural productivity and further threatening livelihoods and food security.^10^ Together, these exposures pose serious risks to community health, increasing the burden of both acute and chronic conditions.

Despite extensive evidence of environmental contamination and ecological disruption in mining-affected areas, a critical gap remains in understanding how these exposures translate into community-level public health impacts.^6^ Existing research and policy responses are largely dominated by environmental science and technical approaches, including biomonitoring, environmental sampling, ecological assessments and economic analyses that prioritise quantifying contaminant levels in air, water, soil and food and linking them to disease outcomes.^11,12^ While essential for characterising exposure magnitude and risk, these approaches offer limited insight into how these hazards are experienced in everyday life, how households adapt to overlapping environmental and socioeconomic pressures or how vulnerability is shaped by poverty, gendered labour roles and land dispossession. As a result, the cumulative public health consequences of gold mining are often examined in isolation from the broader social and governance contexts in which exposure occurs.

At the same time, institutional and stakeholder responses to mining-related health risks remain poorly synthesised. Although Ghana has an extensive regulatory and policy framework governing mining and environmental protection, implementation is uneven, particularly in ASM-dominated settings, and the effectiveness, equity and sustainability of corporate, governmental and community-led interventions are rarely evaluated.^13^ This fragmented evidence base limits understanding of how household adaptive strategies and institutional actions interact, constraining the development of coordinated, intersectoral public health responses capable of reducing exposure and addressing the structural drivers of mining-related health risks.

Against this background, this review consolidates evidence on the public health implications of gold mining in Ghana, with attention to community-reported health and well-being outcomes, household adaptive responses to environmental and socioeconomic disruption and gaps in stakeholder interventions. By centring lived experiences within their environmental and governance contexts, the review highlights how mining-related exposures accumulate and interact with livelihood insecurity and social vulnerability to shape health risks. The findings underscore the need for coordinated, intersectoral approaches that move beyond technical exposure control to address the broader determinants of health in mining-affected communities. These insights are relevant not only to Ghana but also to other resource-dependent settings across the Global South where similar dynamics continue to intensify environmental health risks.

## Methods

### Study design

This scoping review aimed to map evidence on the health and well-being impacts of gold mining in Ghana, the coping strategies adopted by affected communities and stakeholder-led interventions. The review followed Arksey and O’Malley’s methodological framework,^14^ enhanced by guidance from the Joanna Briggs Institute (JBI) Manual for Evidence Synthesis.^15^ Reporting adheres to the Preferred Reporting Items for Systematic Reviews and Meta-Analyses-Scoping Review (PRISMA-ScR) checklist.^16^ No protocol was registered for this review, consistent with common practice for scoping studies; however, all procedures were prospectively planned and documented to ensure transparency and reproducibility.

### Eligibility criteria

Eligibility criteria were defined using the Population-Concept–Context (PCC) framework recommended by JBI.

**Population:** Included studies involving community members living near gold mining operations, miners, other exposed individuals and relevant local stakeholders. Studies focusing solely on representatives of mining companies were excluded.

#### Concept

Included studies reporting health or well-being outcomes associated with gold mining, whether through direct measurement or community perceptions. Excluded were studies examining only economic, environmental or policy impacts without linking them to health, as well as studies based exclusively on laboratory or environmental samples without interaction with affected populations.

#### Context

Included studies conducted in Ghana examining artisanal, small-scale or large-scale gold mining. Studies outside Ghana were excluded.

#### Study types and timeframe

Empirical qualitative, quantitative and mixed-methods studies published between January 2012 and April 2025 were eligible for inclusion. This period corresponds to an era of intensified expansion of both large-scale and artisanal and small-scale gold mining in Ghana, marked by heightened media scrutiny, public debate and political attention to the environmental and public health consequences of mining, particularly illegal galamsey activities. Opinion pieces, reviews and non-empirical reports were excluded.

### Search terms and strategies

A comprehensive search strategy was developed iteratively with support from an academic librarian. Searches were conducted in PubMed, MEDLINE (Ovid) and Scopus, using controlled vocabulary and free-text terms related to ‘gold mining’, ‘health’, ‘community’ and ‘Ghana’. Full database-specific search strings are provided in Online Appendix 1.

To enhance coverage, we searched university repositories (University of Mines and Technology; Kwame Nkrumah University of Science and Technology) and relevant grey literature sources. Grey literature was screened using the same eligibility criteria as peer-reviewed articles. No language restrictions were applied, apart from those inherent to database indexing.

### Selection of sources of evidence

All records were imported into Excel for management. After duplicate removal, screening proceeded in two stages:

- **Title and abstract screening:** Conducted by the lead reviewer, with 20% independently reviewed by a second reviewer to ensure consistency.
- **Full-text screening:** Conducted independently by both reviewers, with disagreements resolved through discussion.

### Data charting

A structured data charting form was developed and iteratively refined to ensure clarity, relevance and alignment with the review’s analytical aims: (1) documenting the health and well-being impacts of gold mining, (2) identifying community coping and adaptive strategies and (3) mapping stakeholder-led response actions. The charting tool captured five domains of information:

- **Study characteristics:** Author(s), year of publication, study location or region, publication type (peer-reviewed article or thesis) and additional bibliographic details.
- **Research approach:** Study aims, design, guiding conceptual or analytical frameworks, participant types, sampling approaches, sample size, data sources (qualitative, quantitative or mixed) and analytical methods.
- **Mining context:** Type of gold mining (artisanal, small-scale, large-scale or illegal [*galamsey*]), operational status, geographic scope and the names of mining companies were reported. Where applicable, the proximate environmental conditions described in the study (e.g. water contamination, dust pollution, land degradation) were also documented to help interpret exposure pathways.
- **Reported impacts:** Populations affected, differential or adverse health outcomes (respiratory, dermatological, waterborne, vector-borne, reproductive, neurological, mental health) and broader well-being effects such as livelihood disruption, food insecurity and social conflict. Where studies provided explicit or implied associations between mining activities, environmental exposures and health outcomes, these were recorded.
- **Community and stakeholder responses:** Community-level coping or adaptive strategies (e.g. livelihood diversification, migration, alternative water sourcing, informal financial support), as well as stakeholder-led interventions including regulatory actions, environmental remediation efforts, health outreach programmes, corporate social responsibility initiatives and identified implementation gaps.

Two reviewers independently charted all included studies and compared entries to ensure accuracy and consistency. Charting was conducted iteratively, with discrepancies identified during comparison and resolved through discussion and re-examination of the full texts. Where disagreements persisted, a consensus was reached through joint review and clarification of coding decisions. The final dataset supported both descriptive mapping of the evidence base and thematic synthesis across the three analytical domains.

### Critical appraisal

Although quality assessment is not required in scoping reviews, the relatively small number of included studies (*n* = 19) and their methodological diversity provided an opportunity to appraise the strength of the evidence. The Mixed-Methods Appraisal Tool^17^ was used because it accommodates the qualitative, quantitative descriptive and mixed-methods designs represented in the review.

All studies were screened using the Mixed Methods Appraisal Tools (MMATs) two preliminary questions and then assessed according to their design-specific criteria: qualitative studies under ‘Methods’ section, quantitative descriptive studies under ‘Discussion’ section and mixed-methods studies under ‘Conclusion and recommendations’ section. Consistent with MMAT guidance, mixed-methods studies were not appraised separately under qualitative or quantitative sections. Instead, they were evaluated on the justification for the mixed-methods design, the quality of integration between components and the coherence of the overall interpretation.

The two reviewers conducted the appraisal independently, recording judgements as ‘Yes’, ‘No’ or ‘Can’t tell’. Discrepancies were resolved through discussion. No studies were excluded based on quality; rather, the appraisal informed the interpretation of findings.

Of the 19 studies, three were qualitative, five were quantitative descriptive and eleven were mixed methods. All qualitative studies met the full MMAT criteria. Quantitative descriptive studies demonstrated acceptable methodological quality overall, but all lacked information needed to judge nonresponse bias, resulting in a consistent ‘Can’t tell’ rating for MMAT item 4.5. Mixed-methods studies showed the greatest variability, but several provided limited detail on how qualitative and quantitative data were integrated or jointly interpreted.

Overall, 11 studies (58%) met 75% – 100% of MMAT criteria and were rated high quality, 7 studies (37%) met 50% – 74% and were rated moderate quality and 1 study (5%) met fewer than 50% and was rated low quality. These findings were used to contextualise the strength and consistency of evidence across thematic categories.

### Data synthesis

A descriptive-analytical approach was used to synthesise findings. Quantitative characteristics (e.g. study design, region, type of mining) were summarised numerically. Qualitative and mixed-methods data were synthesised thematically using a hybrid deductive–inductive approach: deductive coding based on the three review objectives and inductive coding to identify emergent patterns. Themes were compared across study designs, population groups and mining contexts to identify convergences, divergences and evidence gaps.

## Review findings

### General overview of included sources of evidence

**FIGURE 1:**
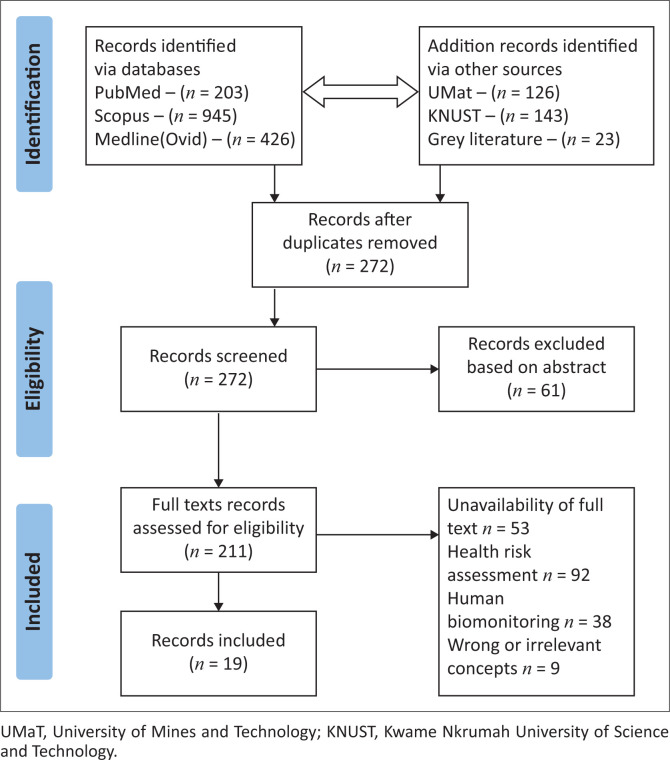
Preferred reporting items for systematic reviews and meta-analyses diagram indicating the selection of included sources of evidence.

The review included 19 studies published between 2012 and 2024, comprising 16 peer-reviewed journal articles and three postgraduate dissertations (Figure 1). Although the search covered studies published between January 2012 and April 2025, no eligible studies published in 2025 met the inclusion criteria at the time of screening.

The included studies employed a range of research designs to examine the health and well-being impacts of gold mining among affected communities in Ghana. Eleven studies used mixed-methods approaches that combined quantitative surveys with qualitative techniques such as in-depth interviews, focus group discussions and document analysis.^7,18,19,20,21,22,23,24,25,26,27^ Five studies adopted cross-sectional quantitative designs to assess outcomes, including malaria prevalence, respiratory diseases and healthcare access in mining-affected communities.^28,29,32^ The remaining three studies used qualitative designs, including case studies and exploratory approaches, to capture community-level experiences such as psychological distress, livelihood disruption and educational impacts.^33,34,35^

Most studies relied primarily on community-based primary data collection. However, two studies^18,19^ incorporated human biomonitoring data, thus integrating biological samples (e.g. blood), environmental assessments (e.g. water samples) and community-level data to triangulate findings on mining-related exposures and health outcomes.

A comprehensive summary of the included studies is provided in Online Appendix 2.

**FIGURE 2:**
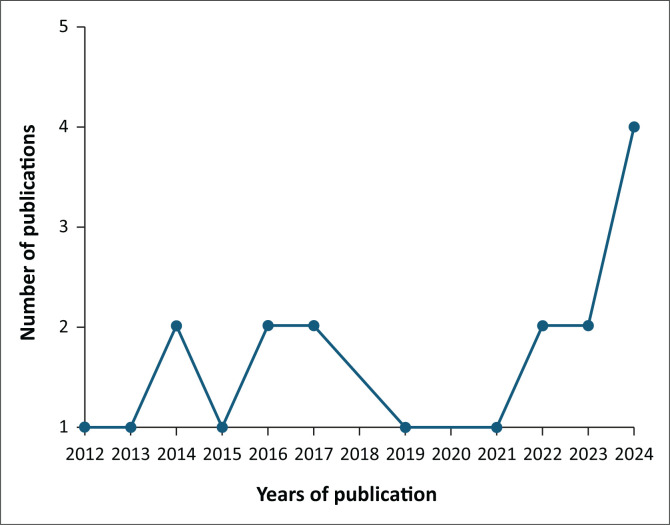
Publication timeline of included studies.

The graph (Figure 2) illustrates a non-linear trend in the volume of publications on gold mining and health over time. Scholarly interest in the topic appears sporadic, with a marked decline in output between 2019 and 2021, potentially attributable to disruptions in research activities during the COVID-19 pandemic. From 2022 onwards, however, there has been a modest resurgence in publications, which may reflect increased public discourse, media coverage and growing academic attention to the health and well-being implications of gold mining in affected communities.

**FIGURE 3:**
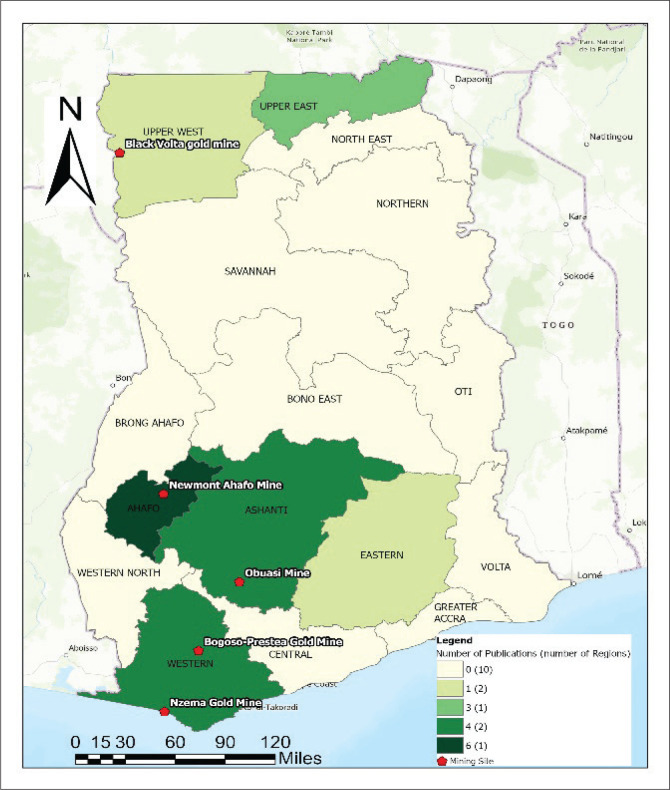
Geographic distribution of included studies.

Out of the 16 administrative regions in Ghana, gold mining activities, both large-scale (LSGM) and artisanal and small-scale (ASGM), are present in multiple regions. However, the included studies captured evidence from only seven regions (Figure 3). Mining-active regions not represented in the included studies were Western North, Central, Bono, Bono East, Savannah, Northeast and Oti.

As shown in Figure 3, the geographical distribution of studies was uneven. The Ahafo Region recorded the highest number of studies (*n* = 6), including four conducted in communities surrounding the Newmont Ahafo Mine (LSGM) and two focused on ASGM contexts. The Ashanti Region contributed four studies, comprising three on ASGM activities and one on the Obuasi Mine (LSGM). The Western Region was also represented by four studies: three examined communities around the Bogoso–Prestea and Nzema mining areas (LSGM), while one focused on ASGM.

In contrast, evidence from northern Ghana remains comparatively sparse. The Upper East Region contributed three studies, all focused on ASGM, while the Upper West and Eastern Regions were each represented by a single study, indicating limited analytical depth and contextual diversity in these settings.

### Differential health outcomes

**FIGURE 4:**
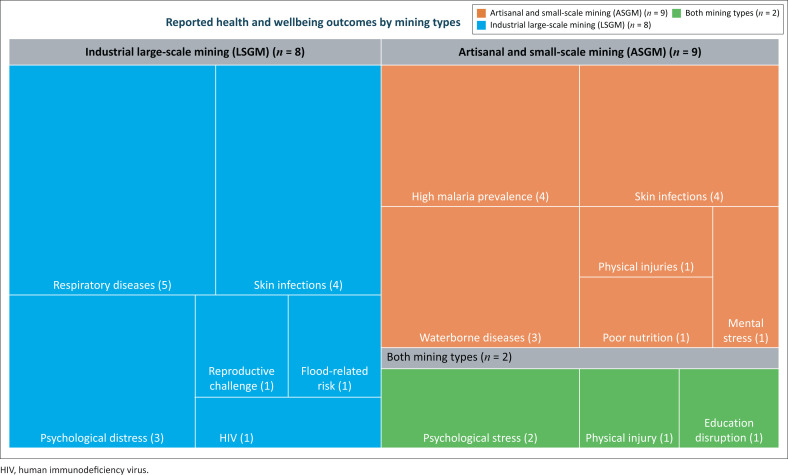
Tree map showing the distribution of reported health outcomes across included studies.

Across the 19 included studies, adverse health and well-being outcomes differed in pattern and emphasis by mining type (Figure 4). While several studies described overlapping environmental exposures, distinct health profiles were evident for artisanal and small-scale gold mining (ASGM) and industrial large-scale gold mining (LSGM). Nine studies examined ASGM,^19,21,23,25,27,29,30,34^ eight focused on LSGM^7,18,20,24,26,28,32,35^ and two included both mining types^31,33^. Because individual studies often reported multiple health outcomes, the counts presented in Figure 4 represent the number of studies reporting each outcome rather than mutually exclusive totals across mining categories.

#### Artisanal and small-scale gold mining

Among the nine studies examining ASGM contexts, malaria was the most frequently reported infectious disease, appearing in four studies^21,22,29,30^ and commonly linked to stagnant water accumulating in abandoned mining pits that created breeding sites for disease vectors. In addition, waterborne infections, particularly cholera, typhoid fever and diarrhoeal diseases, were described in three studies,^23,27,34^ with these conditions associated with contamination of surface and groundwater sources used for drinking and domestic purposes in mining communities. Dermatological conditions were likewise reported in four studies,^22,27,30,34^ as community members described skin irritation, rashes and inflammatory reactions attributed to direct contact with polluted water, contaminated soil and mining residues during extraction and processing activities. Less frequently documented outcomes included physical injuries related to mining activities,^30^ reduced dietary diversity linked to the loss of agricultural land previously used for peasant farming^25^ and psychosocial stress, including depression and anxiety associated with livelihood instability and income uncertainty, particularly among youth,^34^ each reported in a single study.

#### Industrial large-scale gold mining

Across the eight studies examining LSGM contexts, respiratory diseases were the most frequently reported health outcome, appearing in five studies^7,18,20,28,32^ and associated with prolonged exposure to dust and airborne pollutants generated during mining operations. Reported conditions included tuberculosis, asthma, respiratory infections and persistent colds. Dermatological conditions were described in four studies,^7,18,28,32^ ranging from rashes and skin irritation to more severe skin disorders, with one study^7^ specifically noting shingles, particularly during the dry season. Psychological distress was reported in two studies,^20,35^ where experiences of displacement, loss of housing and economic insecurity were associated with feelings of hopelessness and perceived injustice among affected community members. Reproductive health complications, including miscarriage, stillbirth and menstrual irregularities, were identified in one study.^18^ Additional outcomes documented in LSGM settings included flood-related risks^26^ and heightened human immunodeficiency virus (HIV) vulnerability within migrant-labour contexts^7^

#### Studies covering both mining types

Two studies^31,33^ examined health and social impacts in settings where both ASGM and LSGM activities were present. Across these contexts, the studies reported psychological stress among affected community members. Additional health and social outcomes were also documented, including respiratory diseases in one study^31^ and physical injuries alongside educational disruption in another.^33^

### Resilience strategies

Out of the 19 studies included in this review, 17^7,18,20,21,22,23,24,25,27,28,29,30,31,32,33,34,35^ explicitly described individual- and community-level coping mechanisms in response to the health, environmental and socioeconomic challenges associated with gold mining. These strategies reflected locally grounded responses to environmental degradation, livelihood disruption and limited institutional support within mining-affected communities. Thematic analysis identified seven dominant resilience strategies. To facilitate comparison across mining contexts, these strategies were further categorised by mining type: ASGM, LSGM or both, as summarised in Table 1.

**TABLE 1:** Resilience and coping strategies.

Resilience strategy	No. of studies	ASGM	LSGM	Both
Community-level efforts	9	6	3	-
Livelihood diversification and economic survival strategies	8	3	4	1
Health-seeking and protective practices	5	2	2	1
Household water adaptation strategies	5	3	2	-
Psychological and social coping mechanisms	5	2	1	2
Relocation and spatial adjustment	2	1	-	1

LSGM, large-scale gold mining; ASGM, artisanal and small-scale gold mining; No., number.

#### Community-level efforts

Community-level environmental management and collective action responses emerged as the most frequently reported resilience strategy, documented in six ASGM^22,23,25,27,30,34^ and three LSGM studies.^20,24,32^ These responses largely focused on mitigating environmental degradation associated with mining activities. Reported initiatives included covering or filling abandoned mining pits to reduce stagnant water accumulation, land reclamation efforts and informal community initiatives to protect or repurpose degraded communal land. Vector control activities were also described, including pesticide spraying and sanitation campaigns aimed at reducing mosquito breeding.^24,32^

In addition to environmental mitigation, some communities adopted collective agricultural adjustments to cope with mining-related disruptions. These included shifting farming activities to days when mining operations were inactive and increasing the use of agrochemicals to compensate for declining soil fertility or labour shortages.^7^ In LSGM settings, collective responses also extended to community mobilisation and advocacy, with youth groups organising peaceful protests and public demonstrations to demand environmental restoration and raise concerns about the impacts of mining activities on local livelihoods.^24^

#### Livelihood diversification and economic survival

This theme captures income-generating and financial coping strategies adopted in response to mining-related livelihood disruption. It was reported across several studies in both ASGM^22,23,27^ and LSGM contexts.^7,20,24,35^ Households frequently shifted away from traditional farming towards petty trade, small businesses and, in some cases, engagement in mining as a short-term survival strategy.^33^ In addition to income diversification, households relied on informal financial support mechanisms, including family remittances, borrowing through social networks or cooperatives and cash allowances derived from land sales.

#### Health-seeking and protective practices

Five of the seventeen studies^18,28,29,30,31^ described preventive and curative responses adopted by community members to address mining-related health risks across both ASGM and LSGM contexts. Preventive measures included the use of mosquito nets, pesticide spraying, sanitation education and other community-level malaria control initiatives.^30,31^ Some individuals also reported undertaking regular health check-ups in response to ongoing exposure to environmental contaminants.^18,29^ Curative responses involved seeking medical treatment for injuries associated with abandoned mining pits and exposure to hazardous chemicals or equipment, alongside reliance on self-medication and informal care practices.^18,29,30^ In anticipation of urgent or unforeseen health needs, some households maintained active health insurance coverage and increased health-related savings, particularly in LSGM settings.^28^

#### Household water adaptation strategies

Three ASGM studies^21,25,30^ and two LSGM studies^7,32^ reported household-level strategies centred on water substitution. In many mining-affected communities where rivers traditionally serve as the primary source of water for domestic use, pollution of these water bodies prompted households to rely on alternative supplies such as sachet water, harvested rainwater, boreholes and hand-dug wells for drinking and cooking. In some ASGM settings, the degradation of rivers and streams also led farmers to rely on water accumulated in mining pits for irrigation.

#### Psychosocial and social coping mechanisms

Five studies^22,23,31,33,35^ across both ASGM and LSGM contexts reported the normalisation of environmental and occupational risks associated with mining activities. This included denying or redefining exposure to hazards and framing such risks as unavoidable aspects of daily life in mining communities.^22,35^ In some cases, this normalisation was reflected in the acceptance of residents, including adolescents, participating in informal mining activities despite the absence of formal training or protective equipment.^31,33^ One study also reported faith-based coping responses, where community members relied on religious beliefs or spiritual reassurance commonly expressed as ‘leaving it to God’ to cope with uncertainty and hardship associated with mining-related challenges.^35^

Furthermore, community interactions, including sharing experiences, discussing common concerns and informal social networking, were reported as sources of mutual support and solidarity.^23^

#### Relocation and spatial adjustment

Two studies^23,31^ reported migration, often described as relocation, as a response to environmental degradation and livelihood disruption associated with mining activities. This involved the movement of households to alternative locations in search of improved economic opportunities or safer living conditions. In the ASGM study,^23^ farming households relocated to access new agricultural land, sometimes supported by cash compensation from mining companies. Both studies also described relocation arrangements made by mining companies involving compensation packages and the development of newly established communities with basic amenities such as schools and health facilities to facilitate resettlements.

### Existing interventions and gaps in implementation

Across the included studies, interventions clustered into four broad themes: (1) corporate-led infrastructure, compensation and livelihood initiatives; (2) government regulatory and oversight mechanisms; (3) environmental management and remediation efforts and (4) community-led and participatory actions. While these interventions addressed immediate needs, gaps were evident in coverage, enforcement, integration and sustainability.

#### Corporate-led infrastructure, compensation and livelihood initiatives

Mining companies (MC) played a central role in delivering social infrastructure and development-oriented interventions.^7,20,24,27^ These included the provision of boreholes, sanitation facilities, schools, police stations, staff housing, electricity and road infrastructure, as well as hospital construction in some resettled communities. In addition, livelihood-oriented initiatives such as oil palm plantations,^7^ the Ahafo Linkages Program (ALP) to promote local business development and alternative livelihood support schemes were reported.^20^ Compensation mechanisms, both cash and in-kind, were also provided by mining companies, although these were frequently described as limited or inadequate, particularly in relation to resettlement and land loss.^23,26,35^ While these interventions improved access to services and economic opportunities, concerns were raised regarding insufficient compensation and uneven benefit distribution.

#### Government regulatory and oversight mechanisms

Government-led interventions were primarily centred on environmental regulation and institutional oversight. These included monitoring of water quality by the Environmental Protection Agency, enforcement of national mining laws and regulatory roles played by the Ministry of Lands and Natural Resources and the Ministry of Food and Agriculture.^19,25,31,34^ Additional accountability mechanisms, such as oversight by the Commission on Human Rights and Administrative Justice (CHRAJ)^33^ and routine health check-ups through health facilities, were also reported.^18,29^ However, these measures were consistently characterised by weak enforcement, limited monitoring capacity and gaps in addressing environmental risks such as air pollution and odour exposure, particularly in ASM contexts.

#### Environmental management and remediation efforts

Interventions targeting environmental impacts included afforestation and revegetation initiatives, efforts to reduce hazardous chemical discharges and measures to minimise noise and vibration from mining activities.^32^ Some community members (CM) also engaged in localised land restoration efforts.^7^ Despite these activities, environmental hazards such as land degradation and water contamination persisted, indicating limitations in the scale, coordination and long-term maintenance of these interventions.

#### Community engagement, awareness and grassroot responses

Community-level interventions were comparatively limited and often under-resourced. These included local education and awareness initiatives,^34^ as well as forms of grassroot mobilisation such as youth group activism (e.g. Movement of Gbane People for Justice [MOGPEJ]) aimed at documenting rights violations and advocating for accountability.^22^ Across studies, community participation in intervention design and implementation remained minimal, with most efforts described as externally driven and consultative rather than participatory.

## Discussion

In recent years, gold mining in Ghana has attracted increased public and political attention, particularly regarding its health and environmental impacts, which may partly explain the rise in related research since 2021. However, the evidence base remains geographically concentrated in historically dominant mining regions where large-scale gold mining (LSGM) and well-established artisanal and small-scale gold mining (ASGM) coexist. This review identified studies from only seven regions, leaving several mining-active areas – including Western North, Central, Bono, Bono East, Savannah, Northeast, and Oti – unrepresented. As a result, the findings of this scoping review predominantly reflect settings where mining is more visible, regulated, and accessible. This limits understanding of how mining-related health and well-being impacts vary across governance, environmental, and community contexts, particularly in regions where ASGM operates with less oversight.

### Differential health outcomes

Most health impacts identified in this review are consistent with evidence from mining regions across sub-Saharan Africa, where poorly regulated extraction activities have been associated with significant public health challenges. Although many studies relied on self-reported symptoms rather than clinical diagnoses or direct environmental measurements, the consistency of reported associations across different mining contexts suggests plausible exposure pathways. For example, findings align with studies from South Africa and Indonesia showing that elevated concentrations of particulate matter and mining-related dust contribute to increased respiratory disease among nearby populations.^36,37^ Similar exposure pathways have been documented in other extractive regions where blasting, ore processing and heavy vehicle traffic generate sustained airborne pollutants linked to chronic cough, respiratory infections and other respiratory symptoms.^38^

Comparable mechanisms are evident for dermatological and water-related diseases. Studies from mining regions in India have linked polluted surface water to both perceived and clinically observed dermatological conditions,^39^ while outbreaks of cutaneous abscesses have been reported among communities living near mining operations in South Africa.^40^ Findings from this review suggest that contact with polluted water sources in mining-affected communities contributes to skin infections and outbreaks of waterborne diseases such as diarrhoea, cholera and typhoid, while abandoned mining pits that accumulate stagnant water create breeding grounds for mosquitoes and increased malaria transmission. Similar exposure pathways have been documented in mining regions such as Tanzania and the Republic of Congo, where excavation activities contaminate surface water and poorly reclaimed pits promote mosquito breeding, increasing the risk of both waterborne and vector-borne diseases.^41^

Conversely, health outcomes such as heightened HIV risk, widely documented in mining regions across sub-Saharan Africa, were largely absent from the studies included in this review. Evidence from multi-country analyses shows that mine openings are associated with increased risky sexual behaviours, including multiple partnerships and unprotected sex, which contribute to higher HIV transmission.^42^ The limited attention to these outcomes in the Ghanaian literature therefore highlights an important research gap, particularly given that labour migration, alcohol availability, transactional sex economies and limited uptake of HIV prevention services are common features of many mining communities.^43^ Future research should examine how these social dynamics shape sexual health risks in Ghanaian mining contexts.

In this review, psychological distress was commonly associated with the loss of farmland, displacement from ancestral land and uncertainty surrounding household livelihoods. By contrast, studies from mining contexts, such as Turkey and Papua New Guinea, often link distress among non-mining residents to rapid social change, community fragmentation and perceived exclusion from the economic benefits of mining.^44,45^ While geographical and sociocultural contexts may partly explain these differences, psychosocial stress in the Ghanaian context appears to arise primarily from direct livelihood disruption and land dispossession, whereas in other mining regions, distress more frequently emerges from perceived inequalities in benefit distribution and the broader social transformations associated with extractive development.

### Resilience strategies and gaps

Communities in mining-affected areas employ a range of reactive and proactive strategies to manage environmental and socioeconomic disruptions. Collective environmental management efforts, such as filling abandoned pits, land reclamation activities, vector control campaigns and community advocacy, illustrate attempts by local actors to mitigate ecological and health risks where formal governance responses are limited. Similar forms of grassroots environmental management have been documented in artisanal mining regions across sub-Saharan Africa.^41,46^ While these initiatives demonstrate local resilience, they also highlight how responsibility for environmental risk management is often shifted onto communities with limited technical and financial capacity.

Alternatively, households frequently turned to petty trading, small-scale businesses and informal financial support networks to cope with declining agricultural productivity and economic uncertainty. While social networks can buffer households against immediate shocks, such strategies often represent short-term coping mechanisms rather than sustainable livelihood transitions. In the absence of broader economic alternatives or livelihood restoration programmes, diversification into precarious informal activities, particularly illegal mining, may reinforce existing cycles of poverty and vulnerability.

Throughout the literature, mining is frequently promoted as a driver of local development through infrastructure provision, including schools, roads, health centres and water facilities. However, recent studies examining the distribution of mining benefits in Ghana indicated that communities continue to experience high poverty, food insecurity, land dispossession and heightened health risks that are disproportionate to the level of development benefits they receive.^47,48^ This disconnect between resource extraction and local well-being reinforces the broader dynamics of the resource curse, where regions rich in natural resources experience persistent socioeconomic vulnerability.

Despite these challenges, the review also identified evidence of proactive health-seeking behaviours among residents. Community members engaged in preventive and curative practices such as using mosquito nets, participating in sanitation initiatives, attending routine health check-ups and seeking treatment for mining-related injuries or illnesses. These behaviours suggest that risk normalisation does not necessarily translate into passive acceptance of health threats; rather, residents continue to take practical steps to manage health risks within constrained environments. Strengthening these existing practices may therefore provide an entry point for designing more locally grounded public health interventions.

### Strengths and weaknesses of existing interventions

Corporate-led initiatives dominate the response landscape, particularly in the provision of infrastructure, social amenities and livelihood programmes, while government efforts are primarily oriented towards regulation and oversight. Similar patterns have been documented in other resource-constrained settings, where mining companies often assume quasi-development roles in affected communities, complementing but not fully substituting for public sector responsibilities. However, as seen in comparable contexts across sub-Saharan Africa and parts of Latin America,^42,44,45,49^ such interventions are frequently fragmented and variably integrated into national systems, which can limit their continuity and long-term effectiveness. At the same time, persistent gaps in regulatory enforcement, especially in ASM-dominated areas, reflect broader structural constraints observed globally, where limited institutional capacity and competing political and economic interests weaken the translation of policy into practice.

A notable gap across the reviewed studies is the limited evidence on community-led action and collective responses, despite increasing public concern around mining-related impacts. In recent years, Ghana has witnessed episodes of peaceful demonstrations and civic mobilisation, often visible through social media, calling for greater environmental accountability and protection of community livelihoods. However, such forms of grassroot engagement were largely absent from the academic literature included in this review. This disconnect points to a broader limitation in the evidence base, where formal research may underrepresent emerging, informal or digitally mediated forms of community action. In contrast, studies from other settings have shown that stronger community participation and locally driven advocacy can play a critical role in shaping more accountable and contextually grounded interventions, underscoring the importance of better capturing these dynamics in future research.

### Strengths and limitations

It is, to our knowledge, that this scoping review is one of the first to systematically synthesise both the documented health effects of gold mining in Ghana and the range of coping and intervention strategies employed at community and institutional levels. By drawing on diverse sources, including peer-reviewed articles, grey literature and university repositories, it captures a broad evidence base that reflects both biomedical outcomes and community perspectives often absent from more technical health risk assessments. Also, using a JBI framework and PRISMA-ScR reporting, with systematic screening and charting, adds rigour and credibility to the review’s findings.

However, the review is not without limitations. The included studies were uneven in methodological quality, with many relying on cross-sectional or descriptive designs, and only a few incorporating robust epidemiological or longitudinal data. This limits the ability to establish causal pathways or assess the magnitude of risks quantitatively. In addition, most studies were geographically concentrated in Ghana’s major mining regions, leaving important gaps in under-researched areas where mining is expanding. The review is also constrained by the potential underreporting of sensitive outcomes, such as reproductive health and mental health impacts, and by the lack of disaggregated analysis by gender and other social markers. These limitations underscore the need for more rigorous, participatory and equity-focused research to complement and extend the insights generated here.

## Conclusion and recommendations

Taken together, these findings show that the health impacts of mining in Ghana are closely intertwined with broader environmental and socioeconomic transformations in affected communities. Environmental degradation, declining agricultural productivity and the loss of traditional livelihoods reinforce poverty cycles and deepen inequalities between households that benefit economically from mining and those that primarily bear its environmental and health costs. These patterns demonstrate that mining-related health risks extend beyond direct exposure pathways to include structural determinants such as livelihood insecurity, weak environmental governance and social exclusion. Future research should focus on underrepresented mining regions and better capture ASGM activities beyond well-studied areas.

In response, addressing these challenges requires coordinated intersectoral action. Firstly, environmental regulatory bodies, particularly the Environmental Protection Agency and Minerals Commission, should be strengthened to enforce routine compliance audits, mandate public reporting of environmental performance and operationalise reclamation bonds to ensure post-mining land restoration. In artisanal and small-scale mining (ASM) contexts, these enforcement strategies should be adapted through closer collaboration with District Assemblies, traditional authorities and community-based monitoring systems to improve oversight in informal settings.

Secondly, the Ministry of Health should integrate environmental risk factors into national health surveillance systems, including the District Health Information Management System (DHIMS2), to improve early detection and response to mining-related health risks. This integration can be operationalised through district-level linkage of environmental monitoring data with Ghana Health Service routine health information systems, supported by joint environmental–health surveillance structures and strengthened primary healthcare capacity for toxic exposure screening and risk communication.

Thirdly, institutionalising community participation is essential to enhance the relevance, accountability and sustainability of interventions. Structured engagement platforms, led by District Assemblies, should ensure the meaningful inclusion of women, youth and marginalised groups in environmental monitoring, decision-making and intervention design. These efforts should be complemented by community-led monitoring initiatives that enable local reporting of environmental and health concerns.

Fourthly, corporate social responsibility (CSR) initiatives should transition from short-term, project-based activities to long-term, performance-based investments aligned with district health and development priorities. This requires multi-year agreements, clearly defined health and environmental indicators and independent evaluation mechanisms to ensure sustained impact and accountability.
